# Exposure to Bovine Leukemia Virus Is Associated with Breast Cancer: A Case-Control Study

**DOI:** 10.1371/journal.pone.0134304

**Published:** 2015-09-02

**Authors:** Gertrude Case Buehring, Hua Min Shen, Hanne M. Jensen, Diana L. Jin, Mark Hudes, Gladys Block

**Affiliations:** 1 Division of Infectious Diseases and Vaccinology, School of Public Health, University of California, Berkeley, California, United States of America; 2 Department of Pathology and Laboratory Medicine, University of California, Davis Medical Center, Sacramento, California, United States of America; 3 Atkins Center for Weight and Health, University of California, Berkeley, California, United States of America; 4 Division of Community Health and Human Development, School of Public Health, University of California, Berkeley, California, United States of America; Penn State University School of Medicine, UNITED STATES

## Abstract

**Background:**

Age, reproductive history, hormones, genetics, and lifestyle are known risk factors for breast cancer, but the agents that initiate cellular changes from normal to malignant are not understood. We previously detected bovine leukemia virus (BLV), a common oncogenic virus of cattle, in the breast epithelium of humans. The objective of this study was to determine whether the presence of BLV DNA in human mammary epithelium is associated with breast cancer.

**Methods:**

This was a case-control study of archival formalin fixed paraffin embedded breast tissues from 239 donors, received 2002–2008 from the Cooperative Human Tissue Network. Case definition as breast cancer versus normal (women with no history of breast cancer) was established through medical records and examination of tissues by an anatomical pathologist. Breast exposure to BLV was determined by in situ-PCR detection of a biomarker, BLV DNA, localized within mammary epithelium.

**Results:**

The frequency of BLV DNA in mammary epithelium from women with breast cancer (59%) was significantly higher than in normal controls (29%) (multiply- adjusted odds ratio = 3.07, confidence interval = 1.66–5.69, p = .0004, attributable risk = 37%). In women with premalignant breast changes the frequency of BLV DNA was intermediate (38%) between that of women with breast cancer and normal controls (p for trend < .001).

**Conclusions:**

Among the specimens in this study, the presence of amplified BLV DNA was significantly associated with breast cancer. The odds ratio magnitude was comparable to those of well-established breast cancer risk factors related to reproductive history, hormones, and lifestyle and was exceeded only by risk factors related to genetics (familial breast cancer), high dose ionizing radiation, and age. These findings have the potential for primary and secondary prevention of breast cancer.

## Introduction

Breast cancer begins as a change within mammary epithelial cells that starts them on an estimated 20–30 year course through stages of early precancerous changes, carcinoma in situ, and ultimately invasive cancer. Hereditary (familial) breast cancer accounts for fewer than 10% of cases [1]. Although risk factors for the remaining 90% of cases have been identified, including age, reproductive history, exogenous hormones, diet, exercise, alcohol consumption and other lifestyle factors, the exact agents responsible for the initial cellular/molecular changes are not fully understood. Ionizing radiation accounts for <1% of non-hereditary (sporadic) breast cancers [2], leaving the initiating agent for at least 90% of breast cancer cases unaccounted for.

Oncogenic viruses are implicated in at least 6 types of human cancer, the most established of which are hepatocellular carcinoma (hepatitis B and C viruses), carcinoma of the uterine cervix (oncogenic human papillomavirus [HPV] types), Burkitt’s lymphoma and nasopharyngeal carcinoma (Epstein-Barr virus), adult T-cell leukemia (human T-cell leukemia virus [HTLV-1]), and Kaposi’s sarcoma (human herpes virus 8) [3,4]. The longstanding interest in viral initiation of breast cancer stems from the mouse model, the only animal breast cancer with a known etiology. Its causative agent, mouse mammary tumor virus (MMTV), is a retrovirus transmitted from mother to pups via milk [5]. Over the past 50 years there has been considerable interest in determining whether MMTV or a closely related human virus might be transmitted via milk and play an important causative role in human breast cancer. Numerous investigations (1970–1985), found no convincing evidence of an MMTV-like virus in human milk or breast tissues. Although the search was revitalized by the advent of PCR and sequencing, results of the more recent investigations have been divergent and no consensus has been reached [6].

Most humans in Western cultures consume more cow’s milk products in a lifetime than they do human milk, which led us to investigate whether a bovine virus might be an initiating agent for breast cancer. The most prevalent oncogenic virus of cattle is bovine leukemia virus (BLV), a deltaretrovirus closely related to HTLV-1. BLV causes bovine leukosis (leukemia/lymphoma) of B cells [7]. Approximately 38% of beef herds, 84% of dairy herds, and 100% of large-scale dairy operation herds in the USA are infected with BLV [8,9]. Fewer than 5% of these cattle develop clinical leukosis, a condition that mandates exclusion from the US market. BLV-infected lymphocytes circulate through the blood of infected cattle. BLV also infects the mammary epithelial cells of cows [10] and infected cells may be found in cow’s milk [10,11], although pasteurization renders BLV noninfectious [12,13].

In a previous study, we tested humans for antibodies against BLV as a first step in determining if humans were BLV-infected. Using immunoblotting and purified recombinant p24 capsid protein as the target antigen, IgG antibodies were detected in 39% of 257 subjects [14].

To extend the serologic findings, we used in situ PCR to analyze human breast tissues, and detected BLV DNA (retrotranscribed from its RNA genome) in 97/219 (44%) specimens [15]. An independent laboratory validated these results on seven representative specimens [15)] DNA sequences derived from five BLV-positive specimens studied in depth were compared with sequences deposited in the National Center for Biotechnological Information (NCBI) GenBank. They matched sequences of BLV and no others [15]. They did not exhibit the unique signature mutation of our positive control cell line [15]. Using a monoclonal antibody, BLV capsid protein (p24) was detected in 6% of the 219 specimens by immunohistochemistry, suggesting that in some humans, BLV might not be in its usual latent state [16], and viral replication could be occurring.

Thus, in a previous study we have shown that BLV DNA and protein were present in some humans, and were localized primarily to mammary epithelium [15], the cell type from which most breast malignancies arise. We describe here an investigation to explore whether BLV in breast tissue is associated with breast cancer. We conducted a case-control study using malignant breast tissue samples from women with a diagnosis of breast cancer as cases, and nonmalignant breast tissue from women with no history of breast cancer as controls, and examined the association of breast cancer status with presence of retrotranscribed BLV DNA in mammary epithelium as a biomarker of exposure. We tested breast tissue specimens from 239 donors using in situ-PCR and targeting *tax*, the BLV genome region coding for the oncogenic protein responsible for malignant transformation [16]. In deltaretroviral genomes *tax* is rarely deleted during disease progression as are the genome regions related to viral replication (*gag*, *pol*, *env)* [16], and *tax* has the most highly conserved sequence of any deltaretroviral genome region [17]. We also tested the specimens for the presence of the BLV p24 capsid protein as a secondary method.

## Methods

### Specimen Acquisition and Evaluation

Coded breast tissues were acquired through the Cooperative Human Tissue Network, a National Cancer Institute supported tissue bank. Specimens were selected without regard to age, race/ancestry, or diagnosis, from a series of archived breast tissue chronologically acquired from females undergoing breast surgery (2002–2008) at participating hospitals in four catchment areas: Alabama, Pennsylvania, Ohio, and California. The majority (94%) of breast tissues from normal controls originated from reduction mammoplasties, whereas those from breast cancer patients were primarily from mastectomies. All donors gave informed consent for research use of their tissues, and the human use protocol was approved by the Committee for the Protection of Human Subjects, University of California, Berkeley. Information available for all donors was age, gender, date and type of surgery, history of invasive breast cancer, pathology of the specimen, and catchment area. The 114 donors categorized as cases had been diagnosed with invasive breast cancer. The 104 donors categorized as controls had normal breast tissue (free of malignant cells and any premalignant changes), and no previous history of invasive breast cancer. The 21 donors categorized as premalignant had tissue changes classified as premalignant [18] e.g. atypical ductal hyperplasia and carcinoma in situ, and no previous history of invasive breast cancer.

Tissues were formalin-fixed and paraffin embedded at the hospital of origin within 24 hours after surgery. Paraffin blocks were sectioned (4–5μm thick) by the Histopathology Reference Laboratory, Hercules, CA. One section from each sample was stained with hematoxylin and eosin as a reference slide, and the rest were mounted unstained on enhanced adherent slides (Fisher Scientific, #12-550-15). Each specimen was examined, the diagnosis confirmed, and the area of each tissue and area occupied by mammary epithelium determined by our pathologist (HMJ). All samples were evaluated after in situ PCR to determine the pathology of the cell types that were BLV-positive. Tissue age (length of time in the formalin-fixed, paraffin-embedded state) was calculated as the difference in months, between the date of surgery and date of performance of in situ-PCR.

**In Situ Polymerase Chain Reaction (IS-PCR)** was used to detect BLV DNA in the tissue samples. It was based on methods described by Nuovo [19], and was used previously to detect BLV DNA in bovine [20], alpaca [21], and human tissues [15]. The positive control was the BLV-infected FLK (fetal lamb kidney) cell line [22], authenticated as a sheep cell line using species specific primers [23]. Primer sequences, below, were from the *tax* region of the BLV genome. The location of the *tax* primers used is shown below in base pair (bp) numbering according to GenBank accession #EF600696.

Forward (bp 7310–7329): ATGTCACCATCGATGCCTGG

Reverse (bp 7423–7404): CATCGGCGGTCCAGTTGATA

Specificity of these primers for BLV was previously demonstrated by sequence alignments (NCBI BLAST) and lack of amplification of representatives of all oncogenic retroviral families, lentiviruses, as well as viruses reported present in human breast tissues (HERV-K, human papillomaviruses 16, 18, and Epstein-Barr virus)[15]. The in situ-PCR procedure involved incorporation of digoxigenin labeled dUTP into DNA within the fixed cells during one round of 30 cycles of PCR amplification. The incorporated label was then detected by anti-digoxigenin antibodies in an avidin-biotin-immunoperoxidase reaction (Roche). The chromagen was diaminobenzidine (Sigma). Outcome measurement was a semi-quantitative light microscopic judgment of brown color density of the cellular reactions (1+- 4+). See online S1 File for further methodology detail.

**Fig 1:**
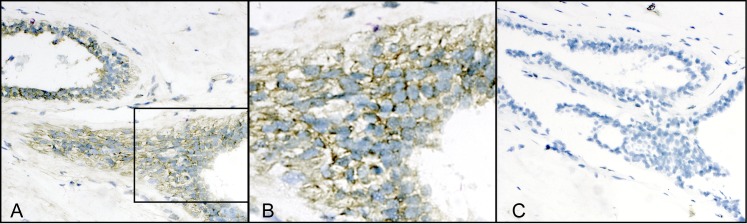
Bovine leukemia virus (BLV) in the mammary epithelium of a donor with breast cancer. (**A)** BLV DNA detected by in situ PCR (brown cells) (X40). Note presence of brown outcome reaction only in epithelium and not in surrounding connective tissue; (**B**) enlargement of boxed area in A, showing that the positive reaction is localized to the cytoplasm of mammary epithelial cells (X136). (**C)** background control, adjacent section reacted with PCR mix without primers to rule out non-specific false positive reactions (X40). Note absence of brown outcome reaction. Counterstain for A,B, and C is Difquick blue.

Specimens were scored positive for the presence of BLV DNA amplicons only if 1) rating of cellular reactions was ≥2; 2) positive and negative cell line controls had the appropriate reactions, pictured in our previous publication [15], which validated the in situ PCR methodology used here; 3) BLV-related amplicons were found in mammary epithelium (Fig 1A and 1B); and 4) the background control for non-specific tissue reactions (an adjacent tissue section reacted with the PCR mix minus primers) was negative for the corresponding area (Fig 1C). Specimens were scored blinded as to donor’s breast cancer status.

**Immunohistochemistry** was performed on formalin fixed deparaffinized tissue sections to identify BLV protein using a monoclonal antibody to the p24 capsid protein in an avidin-biotin-immunoperoxidase reaction. See online S1 File for the detailed procedure.

### Statistical Analysis

Baseline differences between cases and controls were examined with chi square and t-test analyses. The association between BLV status and breast cancer was examined using unconditional multivariate logistic regression [24]. Adjusted models for the full sample included age, race/ancestry, and catchment area. We examined potential effect modification by donor age, race/ancestry and catchment area by including cross-product terms in the logistic model. Donors whose ancestry was unknown were combined with the Caucasian category because most donors from the same catchment area were of Caucasian ancestry. Area of mammary epithelium and duration as fixed samples were highly skewed and no transformation improved skewness. These variables were largely bimodal, with 90% or more of the samples falling in one group, and were examined in stratified analyses. Fit of the model was assessed using the Hosmer-Lemeshow Goodness of fit test [25]. Attributable risk was calculated as: [(proportion exposed among non-cases)(OR-1)]/[(proportion exposed among non-cases)(OR-1) + 1] [24]

## Results

Characteristics of the 218 breast cancer cases and controls are shown in Table 1. Cases were significantly older, by four years at the median, and were predominantly of Caucasian ancestry. Both the amount of mammary epithelium and the length of time in a formalin-fixed state were greater among cases than controls. However, overall, 90% of all specimens had mammary epithelium of 36 mm^2^ or less, and 93% of tissues had been in formalin-fixed state for fewer than 50 months (mean 10.8, SD 12.8 months). BLV positivity was strongly associated with race/ethnicity, and associated to a lesser degree with the other variables.

**Table 1:** Baseline characteristics of breast cancer cases and normal controls.

	All Subjects (n = 218)	Cases (n = 114)	Controls (n = 104)	p^1^	Subjects BLV + (n = 97)	Subjects BLV–(n = 121)	p^2^
Age				< .001			.04
Mean (SD)	52.48(13.28)	55.9(14.1)	48.7(11.2)		54.6(13.89)	50.80(12.57)	
Median	51.00	53.0	49.0		52.0	50.0	
Race^3^				.14			< .001
Caucasian or unknown	157 (72.02)^4^	87 (76.32)	70 (67.31)		79 (81.44)	78 (64.46)	
Other	61 (27.98)	27 (23.68)	34 (32.69)		18 (18.56)	43 (35.54)	
Catchment area				.086			.03
Alabama	80 (36.70)	47 (41.23)	33 (31.73)		32 (32.99)	48 (39.67)	
Pennsylvania	103 (47.25)	47 (41.23)	56 (53.85)		45 (46.39)	58 (47.93)	
Ohio	15 (6.88)	6 (5.26)	9 (8.65)		5 (5.15)	10 (8.26)	
Oakland, CA	20 (9.17)	14 (12.28)	6 (5.77)		15 (15.46)	5 (4.13)	
Tissue age^5^				.17			.04
> 50 months	16 (7.34)	11 (9.65)	5 (4.81)		11 (11.34)	5 (4.13)	
≤ 50 months	202 (92.66)	103 (90.35)	99 (95.19)		86 (88.66)	116 (95.87)	
Area of epithelium^6^				< .001			.44
>36 sq.mm	21 (9.63)	20 (17.54)	1 (0.96)		11 (11.34)	10 (8.26)	
≤ 36 sq.mm	197 (90.37)	94 (82.46)	103 (99.04)		86 (88.66)	111 (91.74)	

Abbreviations: n = number; SD = standard deviation, sq. = square.

^1^Significance of difference between cases and controls.

^2^Significance of difference between BLV+ and BLV-.

^3^Race/ancestry—patient’s self-classification in medical records. ‘Other’ includes 56 African Americans, 3 Asian Americans, 2 other race.

^4^number of subjects (% of total in case and control categories).

^5^Tissue age—the time between initial formalin fixation and the performance of the in situ PCR assay.

^6^Area of epithelium–total area of mammary epithelium present in the breast tissue specimen.

**Fig 2:**
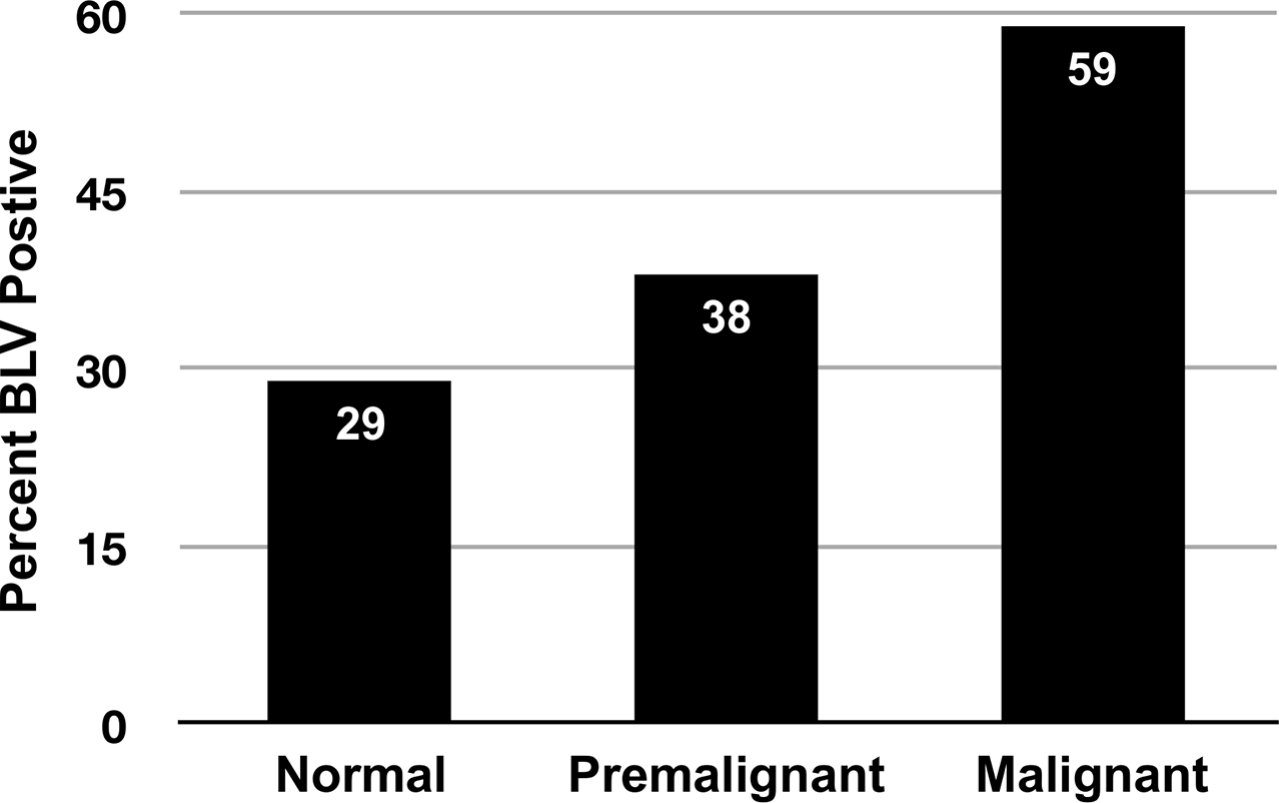
Presence of BLV in mammary epithelium, by diagnostic status. **Normal**: No evidence of malignant or premalignant cells in specimen, and donor has no history of breast cancer; n = 104, SE (standard error) 4.4. **Premalignant:** Specimen contains cells with premalignant changes but no malignant changes, and donor has no history of breast cancer; n = 21, SE 10.6. **Malignant:** Specimen contains malignant cells, and donor has a clinical diagnosis of breast cancer; n = 114, SE 4.6. p for trend < .001.

Presence of BLV-related DNA in breast tissue specimens was strongly associated with breast cancer (multiply-adjusted odds ratio = 3.07, confidence interval = 1.66–5.69, p = .0004, attributable risk = 37%) (Table 2). BLV was detected in the mammary epithelium of 59% of women diagnosed with breast cancer versus 29% of those with no history of breast cancer. BLV frequency in breast epithelium of women with premalignant changes (n = 21) was 38%, intermediate between the frequency in breast cancer cases and normal breast cancer-free controls (Fig 2).

**Table 2:** Association between breast cancer and exposure to bovine leukemia virus.

				Unadjusted		Adjusted^1^	
		Cases	Controls	OR (95% CI)	p	OR (95% CI)	p
All subjects		n = 114	n = 104				
	BLV +	67(59%)	30(29%)	3.52 (2.00–6.19)	< .0001	3.07 (1.66–5.69)	.0004
	BLV -	47(41%)	74(71%)	1.00^2^		1.00	
African American		n = 23	n = 33				
	BLV +	9(39%)	7(21%)	2.39 (0.73–7.79)	.14	1.87 (0.50–7.03)	.35
	BLV -	14(61%)	26(79%)	1.00		1.00	
Caucasian/ Unknown		n = 87	n = 70				
	BLV +	56(64%)	23(33%)	3.69 (1.90–7.17)	< .0001	3.71 (1.80–7.61)	.0004
	BLV -	31(36%)	47(67%)	1.00		1.00	

^1^Logistic regression. Adjusted models include age, race and catchment area for All Subjects; age and catchment for Caucasian or Unknown; and only age for African Americans due to the small sample size. Further adjustment of All Subjects for large area of mammary epithelium or long-term storage increased the odds ratio.

^2^BLV negative subjects are the referent.

Women of African ancestry had an overall frequency of breast tissue BLV of 29% (16/56), significantly lower (p < .01) than the BLV frequency in women of Caucasian ancestry (50%, 79/157) (Table 2). The association of BLV presence with breast cancer was weaker for women of African ancestry (OR = 1.87, CI = 0.50–7.03) than for women of Caucasian ancestry (OR = 3.71, CI = 1.80–7.61), and not statistically significant.

We conducted subgroup analyses to explore the possibility of uncontrolled confounding. Because cancer involves excessive cell proliferation, it was not surprising that some case samples contained larger areas of mammary epithelium than was found in most controls. The odds ratio among those with large samples could not be estimated, since almost all were cases. However, when we examined just the 90% of subjects with mammary epithelium of 36 mm^2^ or less, the odds ratio for the association of presence of BLV DNA with breast cancer was 3.67 (95% CI 1.89–7.13), larger than but consistent with the odds ratio observed among all subjects, and suggesting that the overall finding was not due to an artifact of the amount of mammary epithelium.

We also examined the potential effect of duration of tissue storage, for which more cases than controls had long-term storage. In the 93% of subjects with tissue storage of fewer than 50 months, the odds ratio was 3.26 (95% CI 1.72–6.16) (data not shown), consistent with the odds ratio observed in the whole group and suggesting that the overall finding was not due to artifacts resulting from storage. Furthermore, an interaction term in the main model was not significant.

We also examined the role of age. Within every decade of age, cases had a higher proportion of subjects with exposure to BLV than did controls. More cases than controls had age >80 years; in the 90% of subjects with age between 30 and 80 years, the odds ratio was 3.36 (95% CI 1.86–6.08), suggesting that age was not an important confounder.

We examined whether differences in catchment area could have influenced the result. Since two catchment areas were too small to conduct analyses separately, we repeated the analyses, each time dropping one of the four catchment areas. Each of the four resulting odds ratios was greater than 3.0 and statistically significant.

Most malignant specimens were moderately and poorly differentiated invasive ductal carcinomas; there were too few specimens in other malignant categories to determine their individual associations with BLV DNA. Of 52 subjects whose tissues contained both malignant and nonmalignant cells or who had paired malignant and nonmalignant samples, 36/52 (69%) demonstrated BLV-related DNA in nonmalignant mammary epithelium.

Among 114 cases, 82 had measurements of estrogen receptor (ER) and/or progesterone receptor (PR) status. BLV-related DNA was found more frequently in ER-positive samples (68%) than in ER-negative samples (50%) (p = .11), and more frequently (74%) in PR-positive than in PR-negative samples (51%) (p = .06). Presence of BLV p24 protein was detected in 12/236 (5%) of specimens. There were too few p24 positive specimens to determine if cases and controls differed significantly in p24 production.

## Discussion

Presence of BLV-DNA in breast tissues was strongly associated with diagnosed and histologically confirmed breast cancer, OR = 3.07. As many as 37% of breast cancer cases may be attributable to BLV exposure. The odds ratio is comparable to that of commonly cited reproductive, hormone, and lifestyle risk factors for non-hereditary (sporadic) breast cancer (OR range = 1.2–2.0), and is exceeded only by risk factors related to genetics (familial breast cancer), high dose ionizing radiation, and age (OR range = 3.6–5.8) [26–31].

A comparison of different racial/ancestry groups was not part of our original experimental design but the conspicuously lower frequency of BLV DNA and breast cancer odds ratio in women of African versus Caucasian ancestry is worth mentioning and is consistent with the lower breast cancer incidence among African Americans after age 40, even though their mortality is higher [32,33]. The number of African American subjects (n = 56) in this study however, was limited and further research is necessary to substantiate these observations.

The finding of BLV-related DNA in breast tissues from 29% of normal women is not surprising considering the long latency period of breast cancer, an estimated 20–30 years from the initiating carcinogenic event(s) to appearance of a clinically detectable tumor [34,35], and the life cycle of deltaretroviruses (human, simian, and bovine leukemia viruses). Other oncogenic retroviral families, alpha, beta (to which MMTV belongs), and gamma, achieve oncogenesis by integration into host genomic DNA with perturbation of host protooncogenes and cellular growth control, a mechanism similar to that of endogenous human retroviruses to which beta and gamma retroviruses are closely related phylogentically [36]. In contrast, deltaretroviruses, which are not closely related to any group of endogenous retroviruses [36], do not have to integrate in order to be oncogenic. BLV and its close relative HTLV-1 are usually found unintegrated in lymphocytes during asymptomatic and premalignant phases of bovine leukosis [37] and human T-cell leukemia [38], respectively. During advanced disease phases, when integration into host cell DNA may occur [37], the integration sites for BLV have been found to be random [39]. The oncogenic Tax proteins of BLV and HTLV-1 have been shown to inhibit base excision DNA repair [40,41], through interaction with checkpoint kinase 2 (Chk2) [42,43]. With the resulting decrease in DNA repair, infected cells could gradually, over the lifetime of the infected individual, accumulate unrepaired mutations caused by normal endogenous oxidative metabolism or by exogenous carcinogens, thereby increasing genomic instability, and potentially leading to malignancy. In this respect, deltaretroviruses are considered complex retroviruses, more closely resembling some oncogenic DNA viruses, e.g. hepatitis B virus and human papilloma virus (HPV), which produce an oncogenic protein coded by their own genome.

A well-accepted somewhat analogous cancer initiation mechanism is that of oncogenic types of HPV, which inhibit p53 function, thereby resulting in gradual accumulation of mutations and increasing genomic instability. Therefore, evidence of BLV in normal breast tissues prior to premalignant and malignant changes would be expected. According to the field cancerization theory, most cancers are rare events, developing over decades within a population of carcinogen-initiated cells widespread in a tissue [44,45]. This pattern is observed in carcinoma of the uterine cervix [46], in which the causative virus (HPV) is found not only in the malignant tissue, but also in premalignant dysplastic areas and in normal tissue adjacent to the malignant tumor. The field theory predicts that biomarkers of risk/causation would precede transformation to malignancy, whereas biomarkers of advanced tumor progression, e.g. changes in chromosomes, estrogen and progesterone receptors, and HER2/neu status, would follow transformation to malignancy.

A recent study of the potential of next generation sequencing (massively parallel sequencing) to identify new viral agents of cancer resulted in data that argued against a viral etiology of human breast cancer based on analysis of transcriptome data [47]. However, since transcriptome data rests upon mRNA transcription, BLV was likely not detected because it is so latent in vivo that any mRNA produced would probably be below a detectable level. In previous studies of BLV-infected lymphocytes circulating in the blood of infected cattle with persistent lymphocytosis (a premalignant state), mRNA transcription was detected in only 1 in 50,000 infected cells [48].

How humans become infected with BLV is not known. Transmission from cattle to humans is plausible, as BLV is widespread in both beef herds and dairy herds [8,9]. Although pasteurization renders the virus non-infectious and presumably thorough cooking of beef also does, many people have drunk raw milk and/or eaten raw or undercooked beef at some point in their life. Breast cancer incidence is markedly higher in countries with high milk consumption [49–52]. Numerous prospective studies on dairy consumption in various defined populations [53], however, including one study that carefully evaluated unpasteurized milk consumption [54], found no significant relationship between cow’s milk consumption and breast cancer incidence. Human to human transmission is also plausible. Milk-borne transmission of BLV from cow to calf occurs naturally and HTLV, the human virus closely related to BLV, is transmitted primarily from nursing mother to child in endemic areas [55]. Epidemiologic studies on human breast milk consumption, however, have not found a significant increase in breast cancer among women who were ever breast-fed as infants compared with those who were never breast fed [56,57]. One potential challenge confronting the elucidation of BLV’s route of transmission to humans is the long agricultural association of humans with cattle, which began over 2,000 years ago, while milk pasteurization in western countries was not standard practice until around 1925 [58]. This would have allowed ample time for BLV to enter the human population and become established, yet still be reentering the human population under certain circumstances. The current reservoir for transmission to humans could, therefore, be cattle, humans, or both.

Strengths of this study include biological plausibility, the fact that a well studied animal model (mouse) demonstrated a viral causation of mammary cancer, and the previous demonstration that humans are, in fact, infected with BLV. Another strength is our observed gradient of breast tissue diagnosis in relation to frequency of BLV exposure: 59% of malignant tissues, 38% of tissues with premalignant changes, and 29% of tissues from normal controls were BLV positive (Fig 2).

There are also limitations. The validity of a case-control study depends on the assumption that the controls arise from the same population as that from which the cases arise. In this study, most case samples came from mastectomies for breast cancer, and most control samples came from reduction mammoplasties, which could represent different underlying populations. There were several geographical locations, and there were different race/ethnic groups, which could represent different underlying populations. While we cannot be sure that there aren’t unappreciated differences, we have gone to considerable lengths to assess the possibility that these or other factors were confounders. The association is unlikely to be an artifact of age differences, because we find the same association among younger women, and higher BLV positivity in cases than controls in each decade of age. It is unlikely to be due to racial differences between cases and controls, because we find the association in the 72% of the sample who are of Caucasian origin. The association is unlikely to be an artifact of the geographic location from which the samples arose, because we find the association in each of the two larger catchment areas, and also when we sequentially drop one of the four catchment areas. The observed association is unlikely to be due to the size of the available tissue sample or the length of tissue storage, because we find the same association in the 90% of cases and controls with comparable tissue area and tissue storage.

It is a limitation of the in situ-PCR method upon which the study is based, that the PCR amplicon generated within the tissue DNA cannot be easily sequenced, as in standard PCR. However, our previous study, in which both in situ and solution PCR were performed on different portions of 5 positive specimens, indicated complete concordance of the two methods and >99% homology of sequenced amplicons with published BLV sequences. Contamination of standard solution PCR reactions with DNA of the positive control was ruled out by the absence of the control cell line’s signature mutation at base pair position 5194 [15]. A further strength of in situ PCR methodology is that molecular contamination cannot occur due to the composition of the PCR reaction mix, which differs from that used for standard solution, and allows amplification only within the cells, not in the PCR reaction mix covering the tissue during the reaction [19].

A case-control study is not conclusive in itself. Validation by other investigators is essential, and a prospective study showing that viral infection preceded detectable cancer development would be desirable to support the idea of a causal association of BLV with breast cancer [59]. Regardless of causality or of how the virus has been acquired by humans, if BLV were substantiated as a risk factor for breast cancer, its detection in breast fluid cells or tissues might serve as a biomarker to identify women at higher risk for developing breast cancer and perhaps warranting closer monitoring leading to early detection and treatment. Also since BLV is a retrovirus, the opportunity presents itself to explore the effectiveness of anti-retroviral therapy in limiting or eliminating BLV before breast cancer could be initiated.

## Supporting Information

S1 File Online Supplementary Methodology Information. (DOCX)
