# Supplementary material for: Exposure to Bovine Leukemia Virus Is Associated with Breast Cancer: A Case-Control Study
Source: PLoS One. 2015 Sep 2;10(9):e0134304. doi: 10.1371/journal.pone.0134304 (PMC4557937; doi:10.1371/journal.pone.0134304)
Supplement: S1 File — (DOCX) [file pone.0134304.s001.docx]

**S1 file. Online Supplementary Methodology Information**

In Situ Polymerase Chain Reaction (IS-PCR). The positive control was the BLV-infected FLK (fetal lamb kidney) cell line [1] authenticated as a sheep cell line using species specific primers [2]. FLK cells were smeared onto a 1sq. cm. area of an enhanced adherence microscope slides and fixed 18 hrs in buffered neutral formalin. To enhance entry of PCR mix into cells, samples were permeabilized by digestion with 2mg/ml pepsin in 0.1N HCl, (40-80 min for tissue sections, 20 min for control cell smears). The reaction was stopped by pepsin inactivation solution (0.1M Tris HCl, 0.1M NaCl, pH 7.4) applied for 1 min., followed by a rinse with Dulbecco’s phosphate buffered saline and 5 min in absolute ethanol. Samples, run in duplicate, were surrounded with a frame seal chamber (Bio-Rad, #SLF 0601), 60μl of PCR mix placed into the chamber, and the plastic cover sealed over the frame. PCR mix composition was 4.0mM MgCl_2_, 0.4mM dNTPs, 1μM primers (Operon), 0.06% bovine serum albumin, 8μM digoxigenin-11-dUTP (dig) (Roche Diagnostic Corp.) and .053U/μl Amplitaq Gold polymerase (Applied Biosystems, #4311806), a Taq polymerase activated only at ≥92°C, thereby reducing false positives from nonspecific DNA repair by Taq at cooler temperatures, e.g. during ramping. Cycling conditions were: 1 cycle of 92°C-10 min; 1 cycle of 91°C-2 min, 53°C-1.5 min; 30 cycles of 91°C-30 sec, 53°C-1.5 min, 69°C-2 min; 1 cycle of 69°C-10 min. Primer sequences, below, were from the BLV *tax* region and their location in base pair (bp) numbering is according to GenBank accession #EF600696:

Forward (bp 7310 - 7329): ATGTCACCATCGATGCCTGG

Reverse (bp 7423 - 7404): CATCGGCGGTCCAGTTGATA

Specificity of these primers was previously demonstrated by sequence alignments (NCBI BLAST) and lack of cross-reactivity with other viruses [3].

The in situ-PCR procedure involved incorporation of digoxigenin labeled dUTP into DNA within the fixed cells during PCR amplification of DNA. The incorporated label was then detected by anti-digoxigenin antibodies in an avidin-biotin-immunoperoxidase reaction (Roche). The chromagen was diaminobenzidine (Sigma). Outcome measurement was a semi-quantitative light microscopic judgment of color density of the cellular reactions (1+ - 4+) with only ratings of ≥2+ counted as positive. Specimens were scored positive only if 1) positive and negative cell line controls had the appropriate reactions; and 2) if BLV-related amplicons were found in mammary epithelium, and 3) the background control for non-specific reactions inherent in the particular tissue (an adjacent tissue section reacted with the PCR mix minus primers) was negative for the corresponding area (Figure 1, B1). Specimens were scored blinded as to donor’s breast cancer status.

**Immunohistochemistry** was performed using formalin fixed cell smears or deparaffinized tissue sections (4-5µ thick) on superadherent microscope slides. Samples were quenched of endogenous peroxide in 1% H_2_O_2_ in methanol for 30 min, rinsed in phosphate buffered saline, and antigens unmasked in 10mM citric acid (pH6.0), 10 min at 100°C for estrogen and progesterone receptors, and in citrate buffer (.1M sodium citrate, .04M citric acid, pH 6.0), 30 min at 95°C for BLV p24. Slides were in individual plastic staining containers to prevent transfer of cell or tissue material among samples. Unmasking was followed by a saline rinse and an avidin-biotin-immunoperoxidase procedure according to manufacturer’s instructions (Vector Laboratories). Primary antibodies (mouse monoclonals) for receptors (Vector Laboratories) were diluted 1:40 in blocking buffer (1.5% fetal horse serum [Cansera, Etiobicoke, ON] in saline). The primary mouse monoclonal antibody against BLV p24 capsid antigen [4] (AIDS Research and Reference Reagent Program, NIH **#**12145) was diluted 1:10 in blocking buffer. The specificity of this antibody for BLV p24 (capsid) antigen was previously confirmed by lack of reactivity with other retroviruses and common chronic viruses of humans (3). The chromagen for final visualization was diaminobenzidine (Sigma). The outcome measurement was semi-quantitative, as described above for IS-PCR. Negative controls were adjacent sections reacted with fresh hybridoma medium in place of primary antibody and at the same dilutions as the monoclonal antibodies. The positive control for BLV p24 was cell line FLK [1], which replicates BLV, and for estrogen and progesterone receptors, the hormone responsive human breast cancer cell line MCF-7 (American Type Culture Collection).

REFERENCES FOR SUPPLEMENTARY INFORMATION

1. Van Der Maaten MJ, Miller JM.  Replication of bovine leukemia virus in monolayer cell cultures.  Bibl Haemato 1975;43:360-362.

2. Cooper JK, Sykes, G, King S, Cottril K, Ivanova NV, Hanner R, et al. Species identification in cell culture : a two-pronged moledcular approach. In Vitro Cell Dev Biol Anim 2007;43-344-51. doi: 10.1007/s11626-007-9060-2

3. Buehring GC, Shen HM, Jensen HM, Choi KY, Sun D, Nuovo G. Bovine leukemia virus DNA in human breast tissue. Emerg Infect Dis 2014;20:772-782. doi:10.3201/eid2005.131298

4. Kramme PM, Thomas CB, Schultz RD. Temporal stability of the virus load of cattle infected with bovine leukemia virus. Vet Immunol Immunopathol 1995;45:347-354. doi .org/10.1016/0165-2127(94)05352-S
